# Supplementary figures and images for: Aspirin inhibits tumor progression and enhances cisplatin sensitivity in epithelial ovarian cancer
Source: PeerJ. 2021 Aug 2;9:e11591. doi: 10.7717/peerj.11591 (PMC8340904; doi:10.7717/peerj.11591)

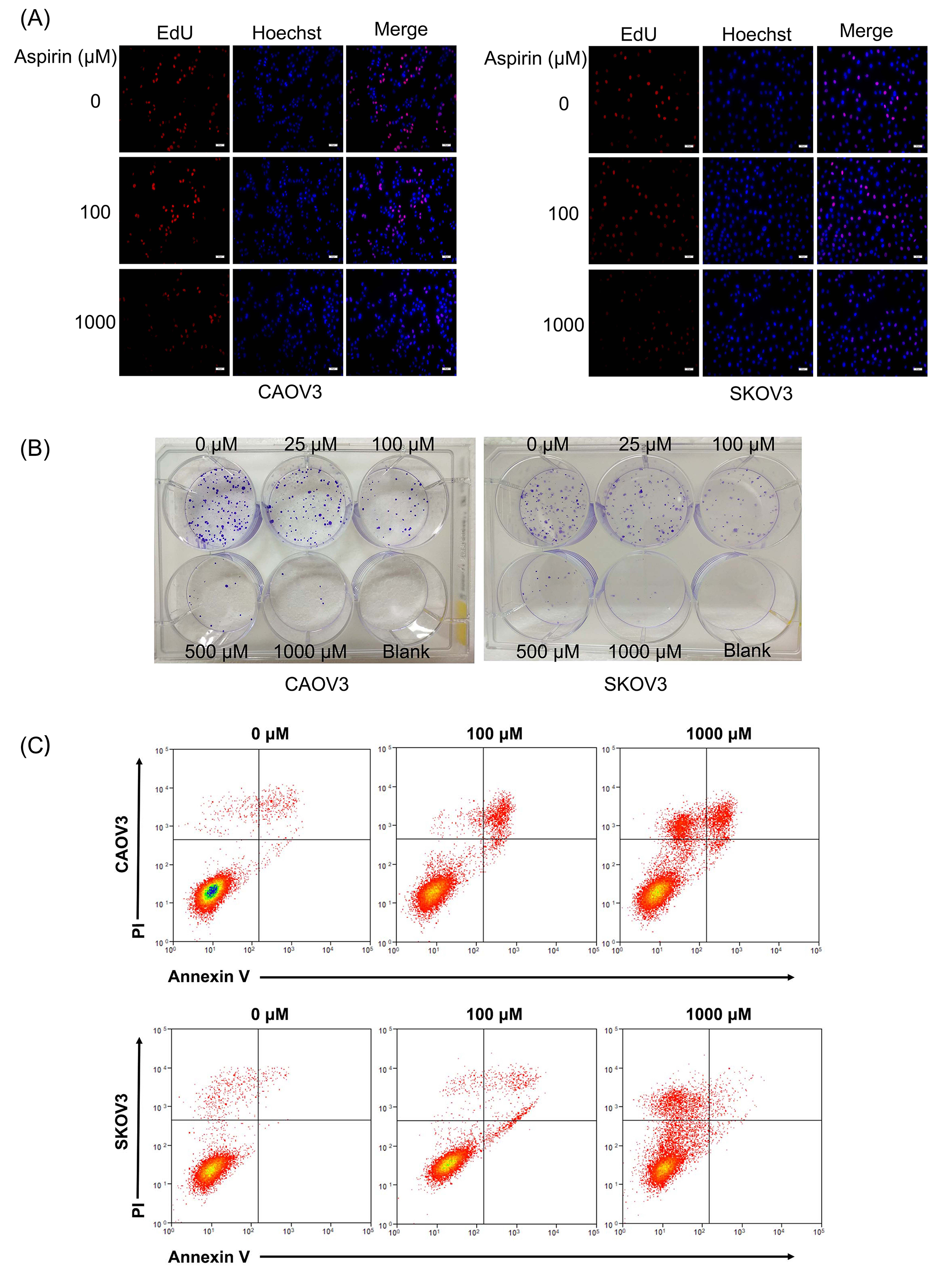

Supplement: Supplemental Information 1 — (A) Cell proliferation was detected using EdU cell proliferation assays (bar, 50 µm) after treatment with different concentrations of aspirin (0 µM, 100 µM, and 1,000 µM) in Caov-3 and SK-OV-3 cells . (B) Colony formation assays were conducted after cells were cultured with different concentrations of aspirin (0 µM, 25 µM, 100 µM, 500 µM, 1,000 µM, and 25 µM) for 14 days in Caov-3 and SK-OV-3 cells. (B) Cells were treated with different concentrations of aspirin (0 µM, 100 µM, and 1,000 µM) for 48 h in Caov-3 and SK-OV-3 cells, stained with the Annexin V-FITC/PI staining kit, and analyzed using flow cytometry. [file peerj-09-11591-s001.png]

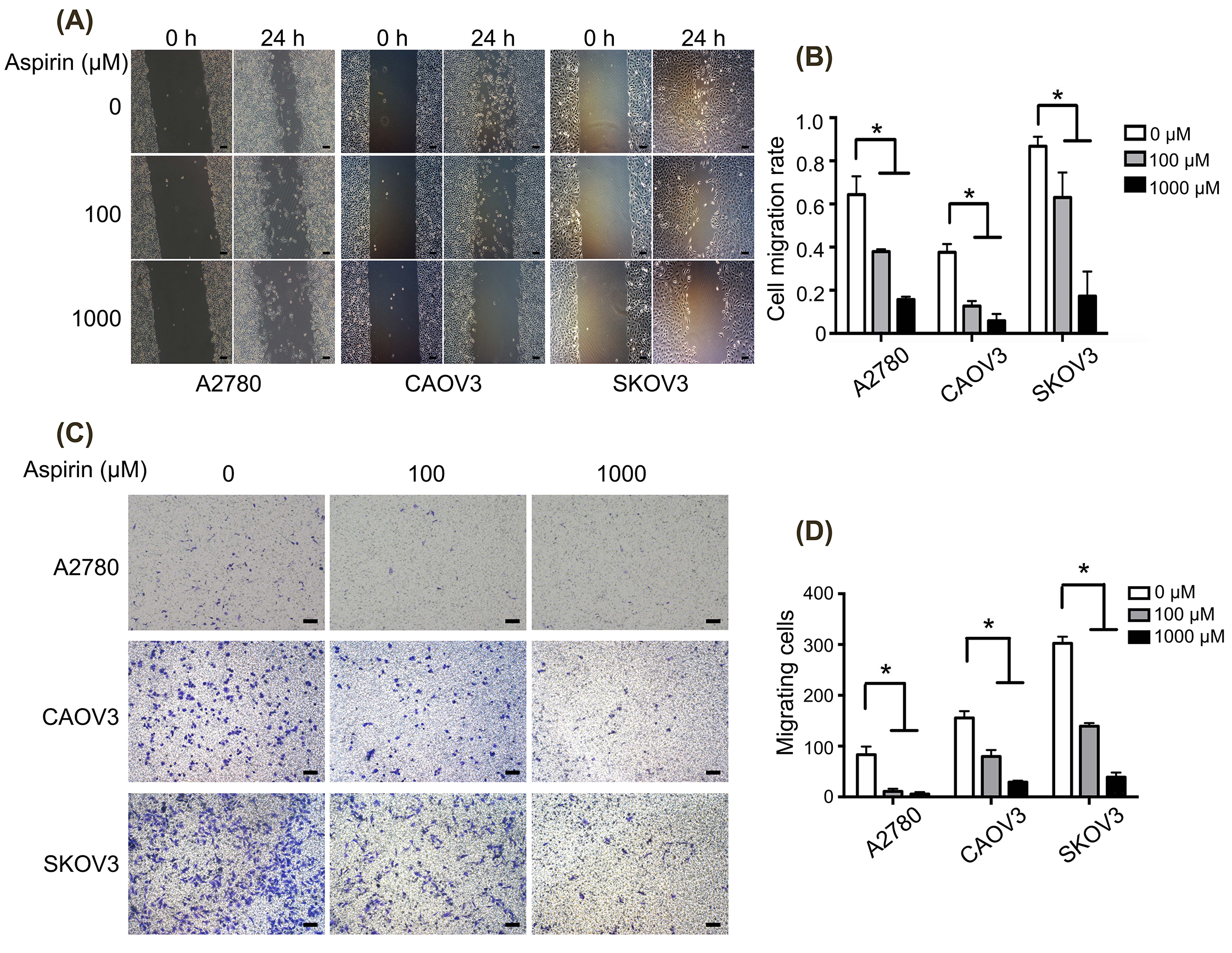

Supplement: Supplemental Information 2 — (A) Wound healing assays were conducted to observe the migration of A2780, Caov-3 and SK-OV-3 cells after treatment with different concentrations of aspirin (0 µM, 100 µM, and 1,000 µM; bar, 100 µm) for 24 h. (B) Quantitative assay of migration rate in A2780, Caov-3 and SK-OV-3 cells. (C) The cell migration rate was determined by performing transwell migration assays after the incubation of A2780, Caov-3 and SK-OV-3 cells with different concentrations of aspirin (0 µM, 100 µM, and 1,000 µM; bar, 100 µm) for 48 h. (D) Quantitative assay of migrating cell counts in A2780, Caov-3 and SK-OV-3 cells. Data are presented as the mean ± SD values. * p < 0.05. [file peerj-09-11591-s002.png]

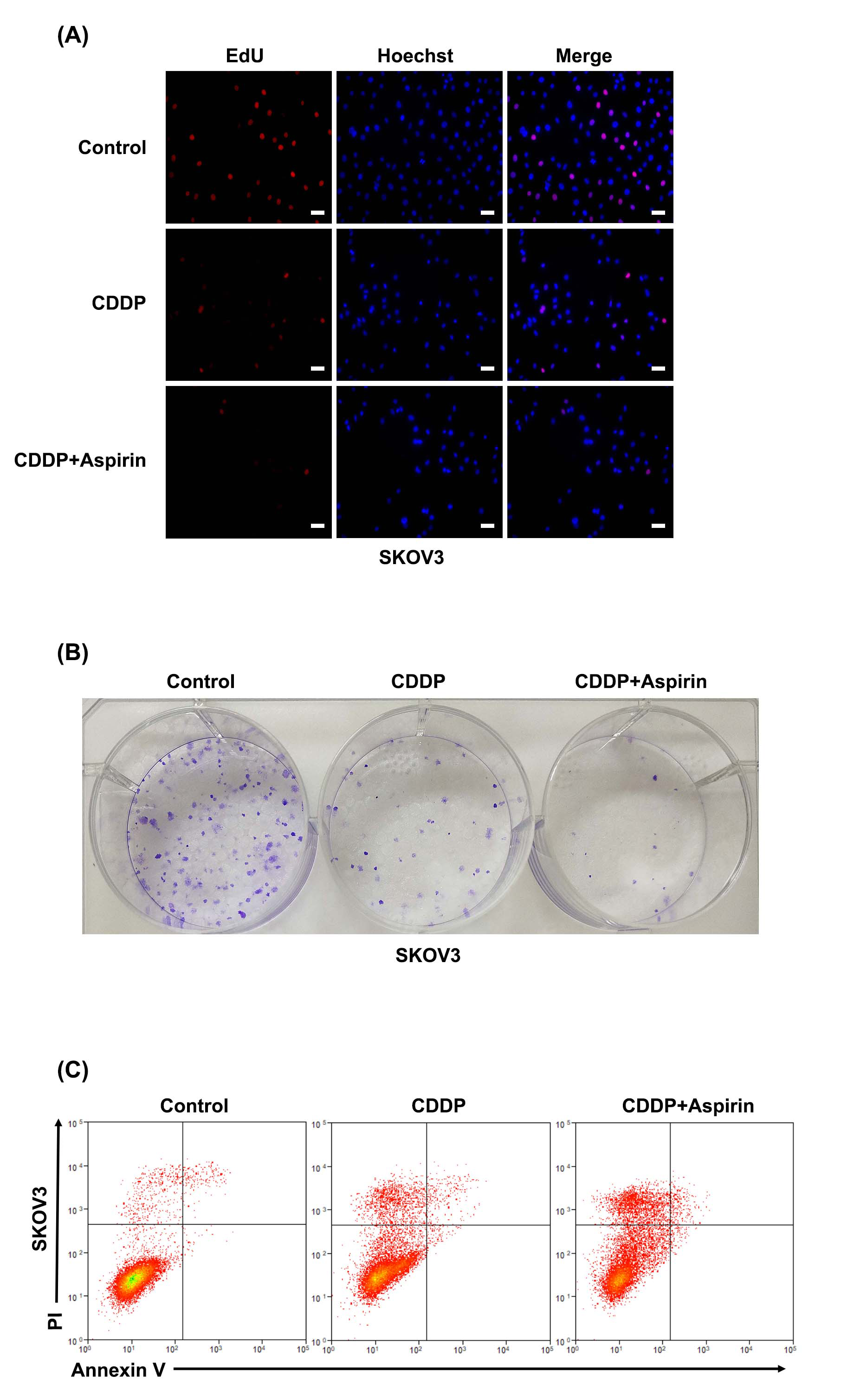

Supplement: Supplemental Information 3 — (A)The cell proliferation rate was determined using EdU cell proliferation assays after the incubation of SK-OV-3 cells with CDDP (20 µM) alone or in combination with aspirin (100 µM) (CDDP + aspirin) for 48 h (bar, 50 µm). (B) Colony formation assays were conducted to observe the growth of SK-OV-3 cells after an incubation with CDDP (5 µM) alone or with aspirin (25 µM) for 48 h. (C) The percentage of apoptotic cells was determined using flow cytometry after the treatment of SK-OV-3 cells with CDDP (20 µM) alone or with aspirin (100 µM). [file peerj-09-11591-s003.png]
